# Exercise Training Can Prevent Cardiac Hypertrophy Induced by Sympathetic Hyperactivity with Modulation of Kallikrein-Kinin Pathway and Angiogenesis

**DOI:** 10.1371/journal.pone.0091017

**Published:** 2014-03-10

**Authors:** José Antônio Silva, Eduardo Tadeu Santana, Martha Trindade Manchini, Ednei Luis Antônio, Danilo Sales Bocalini, José Eduardo Krieger, Paulo José Ferreira Tucci, Andrey Jorge Serra

**Affiliations:** 1 Universidade Nove de Julho (Uninove), Programa de Pós-graduação em Ciências da Reabilitação, Rua Vergueiro, São Paulo, SP, Brazil; 2 Universidade Federal de São Paulo (Unifesp), Rua Napoleão de Barros, São Paulo, SP, Brazil; 3 Universidade de São Paulo, Incor. Av. Dr. Enéas de Carvalho Aguiar, São Paulo, SP, Brazil; 4 Universidade Nove de Julho (Uninove), Programa de Pós-graduação em Medicina, Rua Vergueiro, São Paulo, SP, Brazil; UAE University, Faculty of Medicine & Health Sciences, United Arab Emirates

## Abstract

Sympathetic hyperactivity induces adverse effects in myocardial. Recent studies have shown that exercise training induces cardioprotection against sympathetic overload; however, relevant mechanisms of this issue remain unclear. We analyzed whether exercise can prevent pathological hypertrophy induced by sympathetic hyperactivity with modulation of the kallikrein-kinin and angiogenesis pathways. Male Wistar rats were assigned to non-trained group that received vehicle; non-trained isoproterenol treated group (Iso, 0.3 mg kg^−1^ day^−1^); and trained group (Iso+Exe) which was subjected to sympathetic hyperactivity with isoproterenol. The Iso rats showed hypertrophy and myocardial dysfunction with reduced force development and relaxation of muscle. The isoproterenol induced severe fibrosis, apoptosis and reduced myocardial capillary. Interestingly, exercise blunted hypertrophy, myocardial dysfunction, fibrosis, apoptosis and capillary decreases. The sympathetic hyperactivity was associated with high abundance of ANF mRNA and β-MHC mRNA, which was significantly attenuated by exercise. The tissue kallikrein was augmented in the Iso+Exe group, and kinin B_1_ receptor mRNA was increased in the Iso group. Moreover, exercise induced an increase of kinin B_2_ receptor mRNA in myocardial. The myocardial content of eNOS, VEGF, VEGF receptor 2, pAkt and Bcl-2 were increased in the Iso+Exe group. Likewise, increased expression of pro-apoptotic Bad in the Iso rats was prevented by prior exercise. Our results represent the first demonstration that exercise can modulate kallikrein-kinin and angiogenesis pathways in the myocardial on sympathetic hyperactivity. These findings suggest that kallikrein-kinin and angiogenesis may have a key role in protecting the heart.

## Introduction

Initial activation of cardiac sympathetic drive is observed in chronic heart failure, and it is followed by increased and generalized sympathetic stimulation [1]. Common consequences of sympathetic hyperactivity are negative effects on the heart, such as injury, hypertrophy, and dysfunction [2], [3].

Exercise training exerts several positive effects on the cardiovascular system, such as improved heart function [4], [5]. Moreover, cardioprotective effects of exercise have been extensively described [6]. It was shown that isoproterenol caused hypertrophy, necrosis, apoptosis, fibrosis, and reduced capillary size in the left ventricle (LV) [7]; interestingly, all negative effects of sympathetic hyperactivity were prevented by exercise. In a previous study, we showed that exercise blunted isoproterenol-induced LV hypertrophy as well as improved myocardial performance. These findings were associated with inhibition of pro-inflammatory cytokines in the myocardium [8].

The kallikrein-kinin system is recognized as an important modulator of the cardiovascular system [9], [10]. Tissue kallikrein, a major member of the ubiquitously expressed kallikrein family, releases kinin (bradykinin and kallidin) from kininogen [11]. Kinins exert their action through two G-protein-coupled receptors, kinin B_1_ and B_2_ receptors [12]. Whereas the kinin B_2_ receptor is constitutively expressed in several tissues and cell lines under physiological conditions, the kinin B_1_ receptor normally has very low expression; however, under pathological conditions, particularly inflammation, the kinin B_1_ receptor is synthesized and expressed *de novo* [12].

As noticed for exercise, cardiac hypertrophy and dysfunction were induced as a result of sympathetic hyperactivity that can be attenuated by kinin [13]. In a transgenic rat model harboring human tissue kallikrein, we found that isoproterenol induced less cardiac hypertrophy as indicated by reduction in markers associated with growth and fibrosis. We also observed that the kinin B_2_ receptor antagonist with icatibant eliminated the cardioprotective effects [13].

Analyzing the occurrence of hypotension as a result of physiological adaptation to exercise, some authors have shown that plasma kallikrein activity and bradykinin content increased after exercise [14]. This finding reveals that the cardioprotective effects of exercise against sympathetic hyperactivity may exist with participation of kallikrein-kinin components. We addressed this issue using a well-established experimental model of sympathetic hyperactivity with isoproterenol.

To evaluate the cardioprotective effect of exercise, rats were subjected to isoproterenol after a previous program of aerobic training. We then evaluated several markers expressed under pathologic hypertrophy, including expression of hypertrophic genes, myocytes ultrastructure and fibrosis, myocardial dysfunction, angiogenesis, and apoptosis.

## Materials and Methods

### Ethics Statement

The investigation was designed in accordance with The Guide for the Care and Use of Laboratory Animals published by the US National Institutes of Health (NIH publication no. 85–23, revised 1996). This experimental protocol conformed to government and institutional animal welfare guidelines and was approved by the official animal ethics committee of the Universidade Nove de Julho, Brazil (Process number: 0015/2012) prior to the execution of the experiments. All surgery was performed under conditions to minimize suffering.

### Animals

Thirty-four male Wistar rats, weighing 160–190 g, were randomly assigned to one of the following groups: Con (*n* = 12), non-trained rats that received vehicle subcutaneously (olive oil, 1 ml); Iso (*n* = 13) non-trained rats that received isoproterenol injections (0.3 mg kg−1 day−1) diluted in 1 ml of olive oil; and Iso+Exe (*n* = 9), trained rats which were subjected to sympathetic hyperactivity with isoproterenol (0.3 mg kg−1 day−1).

### Exercise training program

The animals were subjected to running on a motor-driven treadmill for 13 weeks as previously reported [7]. Briefly, animals were made to run on a treadmill for 1 h per day, 6 days per week. The treadmill speed was set at 18 m/min for the first 30 min and was increased to 22 m/min for the remaining 30 min of exercise. The rats were preconditioned to treadmill running for 12 consecutive days before main protocol. The treadmill speed was progressively increased by 3 m/min every 2 days until the final speed of 18 m/min was reached. The sessions initially lasted for 5 min and were increased by 5 min each day to reach 60 min on day 12. The isoproterenol or olive oil was administered on the last day of week 12 and on all seven days of week 13 of exercise, to achieve 8 days of treatment. Twenty-four hours after the last exercise session, rats were anesthetized (overdose urethane: 4.8 g/kg i.p.) and sacrificed.

### Myocardial mass, nuclear volume and hypertrophic genes

The LV was rapidly excised after euthanasia, washed, and whole LV mass was recorded. The LV was fixed in 10% neutral buffered formalin, embedded in paraffin, sectioned in 7 µm thickness, and stained with haematoxylin–eosin according to standard protocols. The nuclear length (major diameter) and width (minor diameter) of longitudinal cardiomyocyte sections were measured on Olympus microscope at 40× magnification. Fifty nuclei from each animal was evaluated and nuclear volume was estimated from the formula for a prolate ellipsoid with Image Tool software 3.0 [7]. Frozen LV was performed as described below for gene expression of atrial natriuretic factor (ANF) and beta-myosin heavy chain (β-MHC).

### Myocardial performance

The myocardial performance was evaluated in posterior papillary muscle removed of LV as described in a previous publication [8]. The muscle were placed in a tissue bath containing modified Krebs–Henseleit solution (mM: 130 NaCl, 5.0 KCl, 1.2 MgCl_2_, 1.5 CaCl_2_, 11 glucose, 20 U insulin and 20 Hepes) bubbled with 100% O_2_ and maintained at 29°C, pH 7.4). The muscles were loaded to contract isometrically at a Grass FTO force transducer (Astro-Med Inc., Grass Instrument Division, West Warwick, RI, USA) and stretched to the apices of their length–tension curves with a micromanipulator (Mitutoyo, model 2046 F, São Paulo, Brazil. The parameters were recorded through the use of AcqKnowledge 3.5.7 software (Biopac Systems Inc.) for determination of peak developed tension (DT), maximal rate of tension increase (+dT/dt) and decrease (−dT/dt). The respective values were normalized as a ratio of the cross-sectional area and papillary muscle mass [15].

### Collagen tissue staining

The LV fixed in 10% neutral buffered formalin was performed as described above. The tissue was stained with picrosirius red and collagen content was analyzed using polarized light observation on Olympus microscope at 40× magnification with Image Tool software 3.0 [7].

### Transmission electronic microscopy

Ultrastructural myocardial evaluation was performed in three rats from each group by electron microscopy. The LV fragments were cut into small 1 mm thick pieces, post-fixed in 1% OsO4 solution for 2 h at 4°C, and then dehydrated and embedded in araldite. Silver or grey thin sections were cut on a Porter- Blum MT-B ultra microtome, mounted on copper grids and stained with uranyl acetate and lead citrate. Preparations were examined through a Philips EM-301 microscope and photographed at 1650× magnification. Five representative microphotographs from each rat were registered to evaluate the capillary numbers per area.

### TUNEL staining

To detect apoptotic cells, a TUNEL assay was performed in 2-cm long, 5-µm thick paraffin embedded, formalin-fixed myocardial sections. Tissue sections were prepared as previously described [7]. The number of TUNEL-positive cells per area was counted using 20× magnification in ten representative microphotographs from each rat.

### Gene expression quantification

To evaluate mRNA, total RNA was extracted from LV with 1 ml of TRIzol reagent (Gibco BRL, Gaithersburg, MD) accordingly to the manufacturer's instructions. One microgram of total RNA was used for cDNA synthesis and Real-Time PCR gene expression analysis. Initially, contaminating DNA was removed using DNase I (Invitrogen) at a concentration of 1 unit/µg RNA in the presence of 20 mM Tris-HCl, pH 8.4, containing 2 mM MgCl_2_for 15 min at 37°C, followed by incubation at 95°C for 5 min for enzyme inactivation. Then, the reverse transcription (RT) was carried out in a 200 µl reaction in the presence of 50 Mm Tris-HCl, pH 8.3, 3 mM MgCl_2_, 10 mM dithiothreitol, 0.5 mM dNTPs, and 50 ng of random primers with 200 units of Moloney murine leukemia virus-reverse transcriptase (Invitrogen). The reactions conditions were: 20°C for 10 min, 42°C for 45 min and 95°C for 5 min. The reaction product was amplified by real time PCR on the 7500 Sequence Detection System (ABI Prism, Applied Biosystems, Foster City, CA, USA) using the SYBRGreen core reaction kit (ABI Prism, Applied Biosystems, Foster City, CA, USA). The thermal cycling conditions were: 50°C for 2 min, then 95°C for 10 min, followed by 40 cycles at 95°C for 15 s and 60°C for 1 min. Experiments were performed in triplicates for each data point. Primers used for realtime PCR were: rat glyceraldehyde 3-phosphate dehydrogenase (GAPDH) forward primer 5′-TGCACCACCAACTGCTTAGC-3′ and reverse primer 5′-GCCCCACGGCCATCA-3′ (GenBank number: NM_017008); rat ANF primers forward 5′- AGCGAGCAGACCGATGAAGC-3′ reverse 5′- GCAGAGTGGGAGAGGTAAGGC- 3′ (GenBank number: M27498.1); rat β-MHC primers forward 5′- CACTCAACGCCAGGA -3′ reverse 5′- TTGACAGAACGCTGTGTCTCCT-3′ (GenBank number: X15939.1); rat kinin B_1_ receptor primers forward 5′-CCTTCCAGGCTTAAACGATTCTC-3′ and reverse 5′-GGTTGGAGG ATTGGAGCTCTAGA-3′ (GenBank number: NM_030851.1); rat kinin B_2_ receptor primers forward 5′-CCACCACGGCCTCTTTCAG-3′ and reverse 5′-CGAACAGCACCCAGAGGAA-3′ (GenBank number: NM_001270713.1); rat tissue kallikrein primers forward 5′- TGTCATCAACAGATACCTCTG-3′ and reverse 5′- GCATGATCTGTCACCATCTGT-3′ (GenBank number: NM_001005382). To access endothelial nitric oxide synthase (eNOS), vascular endothelial growth factor (VEGF) and VEGF receptor 2 mRNA quantification: rat eNOS forward 5′-TGCTGCCCGAGATATCTTCAGT-3′ and reverse 5′-GGCTGCCTTTTTCCAGTT GTTC-3′ (GenBank number: AJ011116), rat VEGF forward 5′-ACAGAAGGGGAGCAGAAAGCCCAT-3′ and reverse 5′-CGCTCTGACCAAG GCTCACAGT-3′ (GenBank number: AF222779.1); rat VEGF receptor 2 primers forward 5′-TGGGGGAGCGTGTCAGAAT-3′ and reverse 5′-CCGCTTTAATTGTGTGATTGAC-3′ (GenBank number: NM_002253). One microliter of RT reaction was used for Real-Time PCR. Target gene mRNA abundance was quantified as a relative value compared with the internal GAPDH reference.

### Western blot analysis

Frozen LV was homogenized in cell lysis buffer (100 mM Tris, pH 7.6, 50 mM NaCl, 10 mM EDTA and 1% Triton X-100) supplied with a proteinase inhibitor cocktail (Sigma Chemical Corp., St Louis, MO, USA). Samples containing 30 *µ*g of the homogenate were subjected to SDS-PAGE in 10% polyacrylamide gels. Separated proteins were transferred onto Hydrophobic Polyvinylidene membranes (Hybond-P, Amersham Biosciences; Piscataway, NJ, USA), and transfer efficiency was monitored with 0.5% Ponceau S staining. Membranes were soaked in a blocking buffer (5% non-fat dry milk, 10 mM Tris–HCl, pH 7.6, 150 mM NaCl and 0.1% Tween 20) for 2 h at room temperature and then incubated overnight at 4°C using specific antibodies: goat anti-kallikrein (1∶500 dilution; Santa Cruz Biotechnology, Santa Cruz, CA, USA); goat anti-VEGF (1∶200 dilution; Abcam, Cambridge, MA, USA); goat anti-VEGFr2 (1∶200 dilution; Abcam, Cambridge, MA, USA); rabbit anti-protein kinase B (Akt, 1∶200 dilution; Santa Cruz Biotechnology, Inc); rabbit anti-phospho(S473)-Akt (1∶200 dilution; Santa Cruz Biotechnology, Inc); mouse anti-B cell lymphoma 2 (Bcl-2, 1∶200 dilution; Santa Cruz Biotechnology, Inc); and rabbit anti-Bcl-2 associated death promoter (Bad,1∶200 dilution; Santa Cruz Biotechnology, Inc.). After incubation, membranes were washed three times and then incubated for 1 h at room temperature with horseradish peroxidase-conjugated secondary antibodies (1∶5000 dilution; Zymed, San Franscisco, CA, USA). The detection was performed with chemiluminescence reagents (Amersham Biosciences, Piscataway, NJ, USA) and values for target protein were normalized to GAPDH.

### Statistical analysis

The Shapiro-Wilk test was used to verify approximately normal statistic distributions. The Levene test was used to assess the equality of variances. One way ANOVA followed by Newman–Keuls test was used to detect differences between groups using GraphPad Prism software (version 4.0, GraphPad Software, Inc, La Jolla, CA, USA). A *p* value <0.05 was considered as significant.

## Results

### Exercise inhibits myocardial hypertrophy induced by isoproterenol

**Figure 1:**
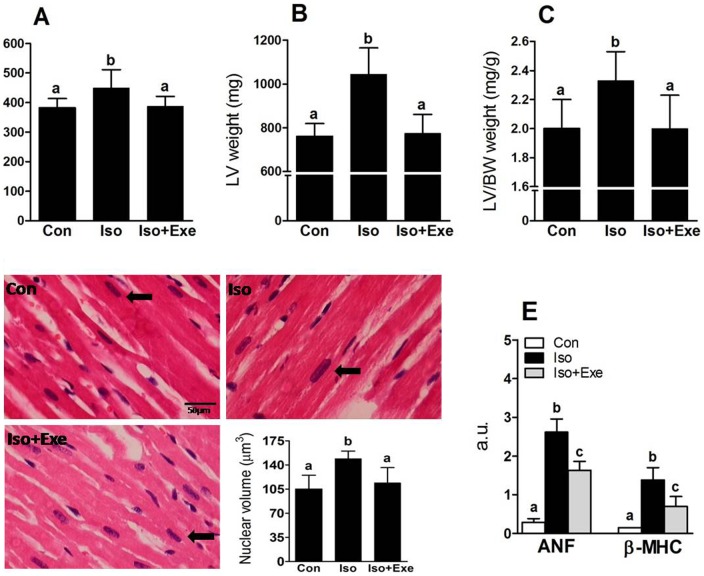
Effects of exercise training on the myocardial hypertrophy induced by sympathetic hyperactivity. Panel **A**, Body weight was evaluated at the end of study. Panel **B**, Absolute left ventricular (LV) mass of each experimental group. Panel **C**, LV mass was indexed by body weight of each animal. Panel **D**, Representative light micrographs of myocardial section stained with haematoxylin–eosin. Arrows indicate the cardiomyocyte nucleus on longitudinal orientation. The graph shows the results for nuclear volume of each experimental group. Panel **E**, Expression of hypertrophic mRNA markers for each experimental group determined by quantitative real-time RT-PCR. Same letters above bars into graphs indicate values not different in ANOVA. Different letters above bars into graphs indicate significant difference between means.

Body weight and LV mass were analyzed for each animal. Sedentary isoproterenol-treated rats had significantly higher body weight compared to the other groups (Figure 1A). In sedentary rats, 8 days of isoproterenol significantly increased LV mass compared with sedentary rats that only received vehicle (Figure 1B/1C). As indicated in Figure 1, there was a clear impact of exercise on LV enlargement; therefore, trained rats showed inhibition of myocardial growth. Since nuclear augmentation is associated with cellular growth [16], LV hypertrophy was confirmed by nuclear volume increase in the Iso group (Figure 1D). Exercise blunted increase in this indicator of cellular hypertrophy.

Pathologic cardiac hypertrophy induced by the isoproterenol model is characterized by the induction of genes normally expressed during fetal development, such as ANF and β-MHC [17]. We evaluated whether exercise prevented the induction of ANF and β-MHC in hypertrophy induced by isoproterenol. Consistent with previous findings, there was increased expression of ANF and β-MHC mRNA in the Iso group (Figure 1D). However, exercised animals expressed significantly less ANF and β-MHC mRNA than sedentary isoproterenol-treated rats.

### Exercise confers myocardial performance protection from isoproterenol

**Figure 2:**
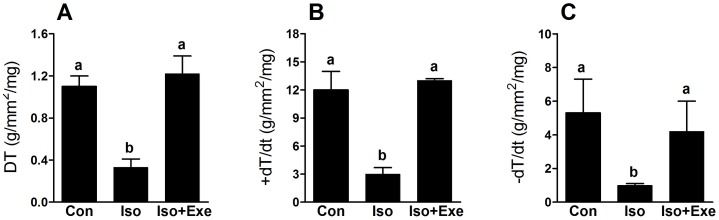
Exercise training inhibits myocardial dysfunction induced by sympathetic hyperactivity. Data were obtained at muscle lengths corresponding to 100% of *L*max. Panel **A**, Peak developed tension (DT). Panel **B**, Maximal positive time derivative of developed tension (+d*T*/d*t*). Panel **C**, Maximal negative time derivative of developed tension (−d*T*/d*t*). Same letters above bars into graphs indicate values not different in ANOVA. Different letters above bars into graphs indicate significant difference between means.

With respect to myocardial performance, we confirmed findings of previous studies in which sustained sympathetic hyperactivity resulted in muscles that developed less force than their respective controls [18], [19]. In our case, the negative effect is depicted as a reduction in DT (Figure 2A) and +dT/dt (Figure 2B). Moreover, −dT/dt (an indicator of myocardial relaxation) was significantly reduced in the sympathetic stimulated non-trained rats compared with non-trained rats that received only vehicle (Figure 2C). Exercised rats subjected to isoproterenol treatment showed that myocardial dysfunction was prevented by exercise.

### There is no expansion of collagen fibers in the myocardia of exercised rats treated with isoproterenol

**Figure 3:**
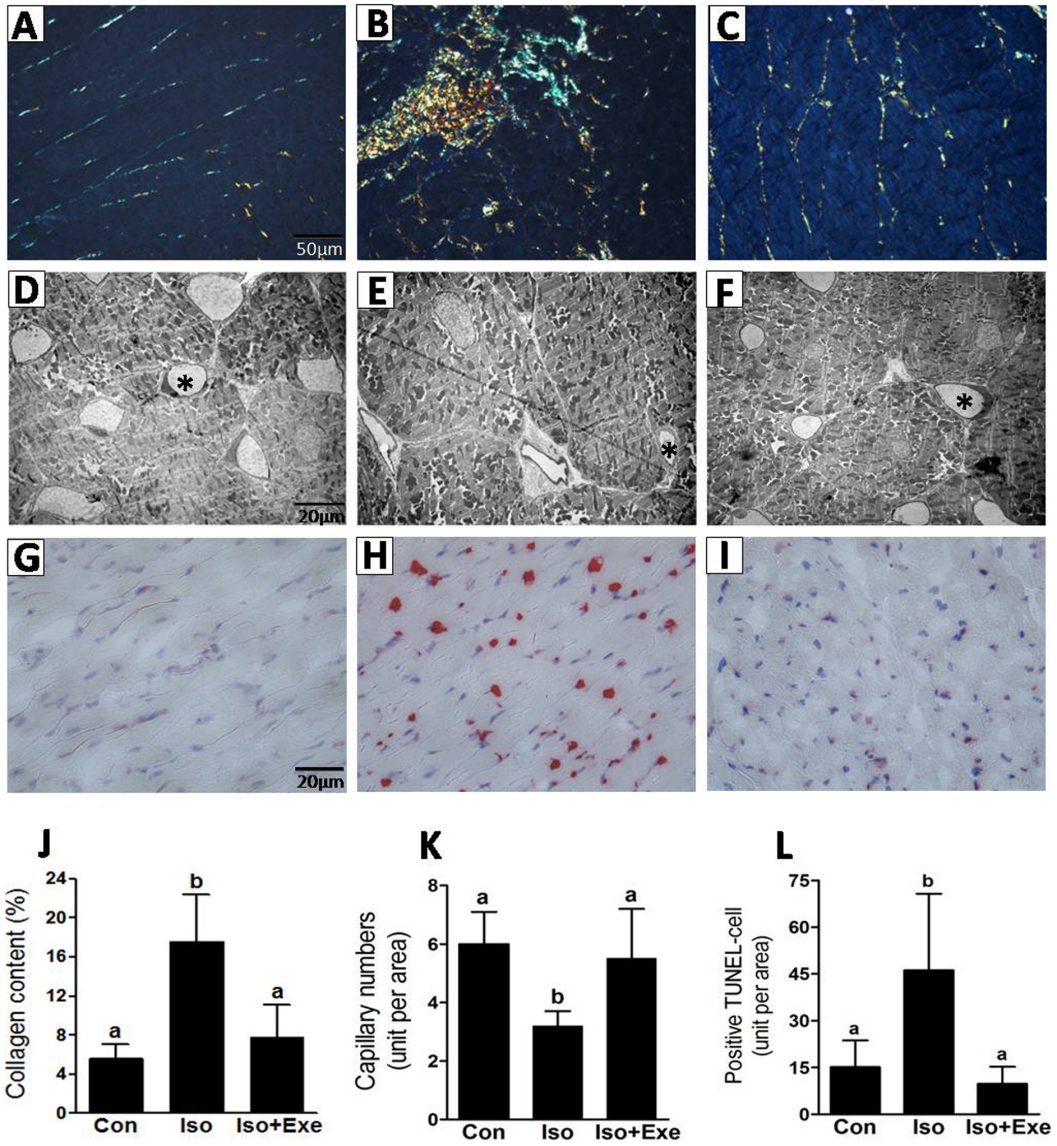
Collagen content, capillary density and apoptosis are preserved in exercised rats on sympathetic hyperactivity. Panel **A–C**, Representative polarized light micrographs of tissue stained with picrosirius red (magnification 40×). Panel **D–E**, Representative electron micrographs for capillaries visualization (magnification 1650×). Panel **G–I**, TUNEL assay for cardiomyocytes in apoptosis (magnification: 20×) as estimated from cells marked in red (magnification 20×). Quantitative analysis for collagen content, capillary density and positive apoptotic cells are shown in panel **J**, **K** and **L**, respectively. Same letters above bars into graphs indicate values not different in ANOVA. Different letters above bars into graphs indicate significant difference between means.

Myocardial fibrosis is a well-established finding associated with isoproterenol-induced sympathetic hyperactivity. Given that the accumulation of collagen has been reported to impair myocardial performance [20], we wanted to test whether exercise could be cardioprotective in cardiac remodeling. As evidenced in Figure 3, quantitative analysis for picrosirius red polarization indicated a significantly larger fractional area of collagen in the Iso group. Notably, isoproterenol treatment showed no discernible effect on collagen content in the LV of exercised animals.

### Capillary reduction and apoptosis are inhibited in exercised rats even after treatment with isoproterenol

It has previously been shown that exercise leads to the preservation of capillaries in the myocardium and may inhibit cell death by apoptosis [7], [8]. As expected, ultrastructural analysis of sedentary isoproterenol-treated rats showed significant reduction of capillaries in the myocardium (Figure 3). Moreover, there was a marked increase in cell death by apoptosis with isoproterenol injections. Interestingly, deleterious effects of sympathetic hyperactivity on capillary and cell death were prevented by exercise.

### Exercise promotes differential expression of kallikrein-kinin components in rats treated with isoproterenol

**Figure 4:**
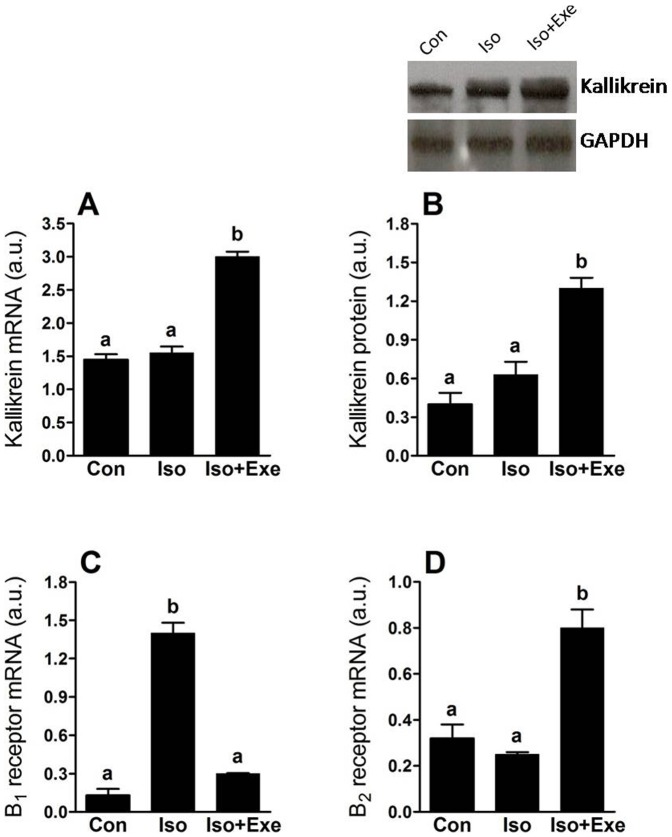
Exercise modulates key components of the kallikrein-kinin pathway in the myocardial on sympathetic hyperactivity. A significantly up-regulation of gene (Panel **A**) and protein (Panel **B**) expression was detected in the trained rats submitted to isoproterenol injection. The kinin B_1_ receptor gene was up-regulated in myocardial of sedentary isoproterenol-treated rats, whereas the exercised isoproterenol-treated rats the kinin B_1_ receptor gene was normalized (Panel **C**). Moreover, exercise also increased kinin B_1_ receptor in transcriptional level (Panel **D**). Same letters above bars into graphs indicate values not different in ANOVA. Different letters above bars into graphs indicate significant difference between means.

The gene expression assay revealed that tissue kallikrein increased significantly in the Iso+Exe group when compared to control animals or those that only received isoproterenol (Figure 4A). There was marked augmentation in tissue kallikrein protein expression of exercised isoproterenol-treated rats when compared with the other groups (Figure 4B). As shown in Figure 3C, sympathetic hyperactivity evoked significant up-regulation of the kinin B_1_ receptor at the transcriptional level. On the other hand, the effects of isoproterenol on kinin B_1_ receptor mRNA were blunted in the exercised animals. Additionally, increased kinin B_2_ receptor expression was observed in the Iso+Exe group compared with the other groups (Figure 4D).

### Exercise modulates different components of angiogenesis and apoptosis pathways

**Figure 5:**
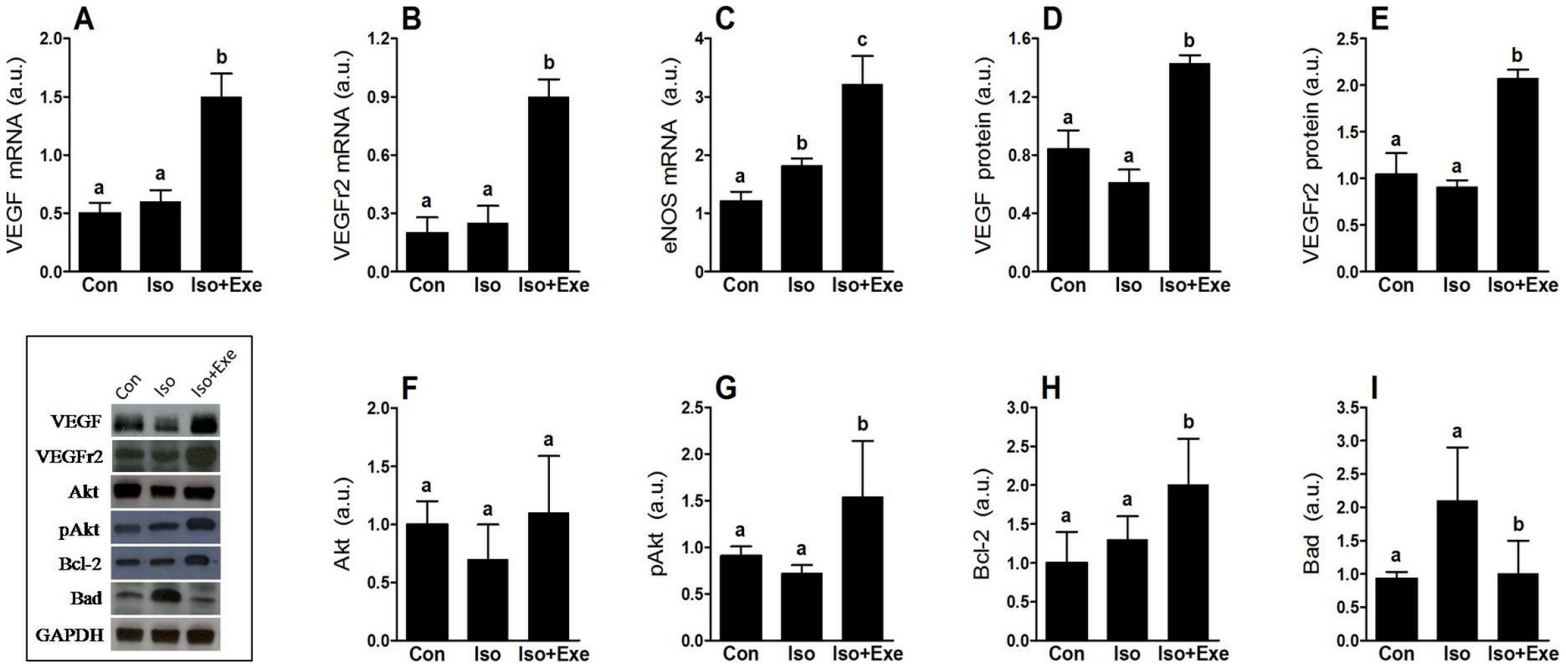
Exercise modulates key components of the angiogenesis and apoptosis pathway in the myocardial on sympathetic hyperactivity. It is remarkable that exercise increased mRNA of VEGF (Panel **A**), VEGF receptor 2 (Panel **B**) and eNOS (Panel **C**) in the isoproterenol-treated rats. The protein levels of VEGF (Panel **D**) and its receptor (Panel **E**) were also increased by exercise. Although total Akt protein has not been changed (Panel **F**), activated form of Akt was significantly up-regulated in the exercise animals (Panel **G**). Moreover, a beneficial effect of exercise was observed for proteins that modulate apoptosis (Panel: **H** and **I**). Same letters above bars into graphs indicate values not different in ANOVA. Different letters above bars into graphs indicate significant difference between means.

Angiogenesis data are shown in the Figure 5. It can be seen that 8 days of sympathetic hyperactivity did not result in change for any component in the non-trained rats. However, we showed significant myocardial up-regulation of VEGF and its type 2 receptor in the trained isoproterenol-treated rats when compared with the Con and Iso groups (Figure 5A–B/5D–E). The eNOS mRNA was evaluated because is usually downstream of kinin and the VEFG pathway (Figure 5C). Thus, gene expression of eNOS increased in the Iso group compared with the Con group. Interestingly, exercise also increased eNOS mRNA content after isoproterenol injections, but its expression was strongly up-regulated compared with the Iso group.

We also observed that another important target of the kinin/VEGF pathway, Akt, was activated by exercise. Hence, total Akt content is not altered by isoproterenol or exercise (Figure 5F); however, its phosphorylated active form (pAkt) showed a significant increase in trained isoproterenol-treated rats (Figure 5G).

Additionally, data for two molecules that are known to modulate apoptosis are shown in Figure 5. Exercise induced a significant increase in anti-apoptotic Bcl-2 protein expression after isoproterenol treatment (Figure 5H). Nevertheless, the increase of pro-apoptotic Bad protein expression evoked by sympathetic overload was blunted by exercise (Figure 5I).

## Discussion

Exercise training is strongly recommended to improve cardiovascular health [21], [22]. Our study was designed to test the hypothesis that cardioprotective effects of exercise on sympathetic hyperactivity are associated with modulation of key components of the kallikrein-kinin system and angiogenesis pathway.

Isoproterenol is well known to induce hypertrophy, fibrosis, and inflammation in the heart when administrated subcutaneously [13], [23], [24]. We previously showed that exercised rats had significant inhibition of deleterious isoproterenol effects [7]. Extension of these findings were published elsewhere, and revealed that the beneficial role of exercise was accomplished by significant improvement in myocardial performance [8]. In this study, there was complete protection from myocardial hypertrophy and dysfunction in rats that received isoproterenol after exercise. Fibrosis, apoptosis, and capillary reduction induced by isoproterenol were also blunted in exercised rats.

Previous findings have raised interest regarding the possible mechanisms mediating the cardioprotective actions of exercise on sympathetic hyperactivity. The prevention of fibrosis, pro-inflammatory cytokines, oxidative stress, and apoptosis is of particular interest [7], [8], [25]. The present study provides novel information regarding this issue. We found that the kallikrein-kinin system was positively modulated in the myocardial of rats on a regular exercise regime. Thus, tissue kallikrein (a protein key for the synthesis of bradykinin) expression at transcriptional and translational levels was augmented. These findings are interesting considering that cytoprotective effects have been linked to kallikrein.

It was shown that protection by tissue kallikrein in oxidative organ damage is attributed to inhibition of apoptosis, inflammation, hypertrophy, and fibrosis [26]. Tissue kallikrein knockout mice showed thinning of the LV wall and reduced myocardial mass compared with wild-type mice. These structural abnormalities were accompanied by reduced cardiac function, which was observed under basal conditions or acute β-adrenergic stimulation [27]. Our findings suggest that tissue kallikrein is possibly participating in prevention of deleterious cardiac effects evoked by sympathetic hyperactivity in exercised rats. Regarding tissue kallikrein expression, the protein analysis corroborates gene expression, indicating that tissue kallikrein is highly formed in the myocardium.

We showed that isoproterenol increased kinin B_1_ receptor mRNA expression, but exercise was able to inhibit this event. Interestingly, B_2_ receptor mRNA modulation only occurred in the exercised animals. There are no data linking deleterious or protective roles of bradykinin receptors in the heart on sympathetic hyperactivity. Therefore, it is difficult to speculate whether exercise-induced cardioprotection may be mediated by the synchronized effect on kinin B_1_ and B_2_ receptors. However, studies indicate a distinct role of these receptors in cardiac remodeling.

Treatment with kinin B_1_ receptor antagonist improved cardiac function after myocardial infarction, as evidenced by attenuation of elevated LV end diastolic pressure [28]. On the other hand, it was shown that tissue kallikrein, through the kinin B_2_ receptor and NO formation, improves cardiac function, apoptosis, and inflammation, and limits LV remodeling after ischemic injury [29], [30]. Additionally, it was shown that B_2_ receptor knockout mice subjected to myocardial infarction had a greater cardiomyocyte cross-sectional area and more interstitial collagen compared with wild-type controls [31].

Studies have suggested a possible angiogenesis therapy using tissue kallikrein based on the fact that human tissue kallikrein was shown to be protective [32]. In our study, we evaluated VEGF expression and its type 2 receptor. We showed that sympathetic hyperactivity does not change VEGF and Akt, which is a key intracellular mediator of this pathway. However, our findings are in accordance with lines of evidence showing that exercise induces a local angiogenic phenotype characterized by overexpression of VEGF in the heart [33]. Moreover, we observed high expression of active Akt form and Bcl-2 (anti-apoptotic) protein as well as a reduction of pro-apoptotic Bad. These findings have been previously shown in myocardial injury by ischemia/reperfusion, hypertension, and diabetes [34], [35], [36]. Thus, as a novel finding, we show that the kallikrein-kinin system/VEGF/Akt pathway may be involved in exercise-induced cardioprotection against sympathetic hyperactivity.

In the current study, one cardioprotective pathway elicited for kinin and VEGF action could be NO release [37], [38]. NO is a short-lived free radical gas involved in several physiological and pathological processes. When synthesized by eNOS, NO plays an important role in endothelial function and cardioprotection [39], [40]. In fact, findings have emphasized that NO may antagonize sympathetic stimulation [41]. Therefore, our findings showed an increase of eNOS in exercise rats, suggesting that this molecule may participate in cytoprotection from the cardiotoxic effects of catecholamines.

### Conclusion

Our results represent the first demonstration that exercise modulates sympathetic hyperactivity in myocardia by the kallikrein-kinin system and angiogenesis pathway. The maintenance of capillarity and prevention of hypertrophy, fibrosis apoptosis, and myocardial dysfunction with exercise are also promising results. Thus, the kallikrein-kinin system and angiogenesis pathway play key roles in protecting the heart from sympathetic stimulation.

### Clinical perspective and limitations

Sympathetic activity increases in a wide range of cardiac diseases, such as ischemic heart failure [42]. Importantly, pronounced sympathetic activation has been shown to be inversely correlated with survival [43]. Our study has important implications regarding this issue. We used an experimental model of sympathetic hyperactivity with isoproterenol to test the protective role of exercise. Hypertrophy, fibrosis, capillary loss, apoptosis, and myocardial dysfunction were prevented by exercise. These findings were accompanied by favorable modulation of components of the kallikrein-kinin and angiogenesis pathways. Moreover, assuming that the isoproterenol load used in our study is also excessive with regard to natural sympathetic stimulation, exercise can be considered very effective for promoting heart protection against sympathetic hyperactivity. Importantly, our rat exercise protocol (1 h per day; 6 days per week; moderate load) was equivalent to human endurance exercise recommendations for heart health, for which moderate-intensity exercise training includes ≥30 min·d^−1^ on 5 d·wk^−1^ for a total of ≥150 min·wk^−1^. In fact, 30–60 min·d^−1^ of moderate exercise has a strong evidence statement (category A) [44]. In the present study, there was a limitation that must be considered. Unfortunately, in the current design, it cannot be determined whether the benefits of exercise training will continue to counteract isoproterenol over longer term exposure, which would be more relevant to chronic human sympathetic hyperactivity.
